# Impact of obstetric interventions on condition at birth in extremely preterm babies: evidence from a national cohort study

**DOI:** 10.1186/s12884-016-1154-y

**Published:** 2016-12-13

**Authors:** Andrei S. Morgan, Neil Marlow, Elizabeth S. Draper, Zarko Alfirević, Enid M. Hennessy, Kate Costeloe

**Affiliations:** 1 Institute for Womens’ Health, UCL, 74 Huntley Street, London, WC1E 6AU UK; 2 Homerton Hospital, London, UK; 3 University of Leicester, Leicester, UK; 4 Institute of Translational Medicine, University of Liverpool, Liverpool, UK; 5 The Wolfson Institute, Queen Mary University of London, London, UK

**Keywords:** Antenatal steroids, Tocolysis, Caesarean section, Extreme prematurity

## Abstract

**Background:**

To investigate perinatal decision-making and the use of obstetric interventions, we examined the effects of antenatal steroids, tocolysis, and delivery mode on birth in a good condition (defined as presence of an infant heart rate >100 at five minutes of age) and delivery-room (DR) death in extremely preterm deliveries.

**Methods:**

Prospective cohort of all singleton births in England in 2006 at 22–26 weeks of gestation where the fetus was alive at the start of labour monitoring or decision to perform caesarean section. Odds ratios adjusted for potential confounders (aOR) were calculated using logistic regression.

**Results:**

One thousand seven hundred twenty two singleton pregnancies were included. 1231 women received antenatal steroids, 437 tocolysis and 356 delivered by Caesarean section. In babies born vaginally, aOR between a partial course of steroids and improved condition at birth was 1.84, 95% CI: 1.20 to 2.82 and, for a complete course, 1.63, 95% CI: 1.08 to 2.47; for DR death, aORs were 0.34 (0.21 to 0.55) and 0.41 (0.26 to 0.64) for partial and complete courses of steroids. No association was seen for steroid use in babies delivered by Caesarean section. Tocolysis was associated with improved condition at birth (aOR 1.45, 95% CI: 1.05 to 2.0) and lower odds of death (aOR 0.48, 95% CI: 0.32 to 0.73). In women without spontaneous labour, Caesarean delivery at ≤24 and 25 weeks was associated with improved condition at birth ((aORs 12.67 (2.79 to 57.60) and 4.94 (1.44 to 16.90), respectively) and lower odds of DR death (aORs 0.03 (0.01 to 0.21) and 0.13 (0.03 to 0.55)). There were no differences at 26 weeks gestation or in women with spontaneous labour.

**Conclusions:**

Antenatal steroids are strongly associated with improved outcomes in babies born vaginally. Tocolysis was associated with improvements in all analyses. Effects persisted after adjustment for perinatal decision-making. However, associations between delivery mode and birth outcomes may be attributable to case selection.

**Electronic supplementary material:**

The online version of this article (doi:10.1186/s12884-016-1154-y) contains supplementary material, which is available to authorized users.

## Background

Any perinatal intervention at extremely preterm gestations may have significant impact on long term health of surviving children. There is often uncertainty over the use of antenatal steroid, tocolysis or mode and timing of delivery due to a paucity of evidence in this population. In certain clinical situations, delaying delivery may be particularly beneficial at such low gestational ages, although emergency Caesarean section may be the only way to prevent stillbirth. There is anxiety that a potentially hostile *in utero* environment may have an immediate or subsequent adverse impact, and any potential benefit of delaying delivery needs to be set against the risk of complications associated with adverse perinatal outcomes [1].

EPICure 2 is a whole population study of extremely preterm births to women resident in England in 2006. Short and long term outcomes have been reported elsewhere [2, 3]. Compared with the original EPICure study in 1995 for births 22–25 weeks, [4] a 13% improvement in survival was demonstrated, but no improvement in the frequencies of major morbidities found [2]. Additionally, evaluation of risk factors at birth in those admitted to neonatal units in 1995 identified the baby born after use of antenatal steroids whose heart rate was greater than 100 beats per minute (bpm) at 5 min after birth as more likely to survive and to have less long term morbidity [4, 5].

In the 2006 cohort, background data were collected about the pregnancy, obstetric management and any antenatal counselling, and overall outcome for all births. This was done *a priori* to determine how antenatal complications, perinatal decision-making and management in labour influence condition at birth in those born before 27 completed weeks gestational age [5]. For babies born alive and admitted to neonatal intensive care, further data were then collected about their condition, treatment and outcome at discharge.

We evaluated the relationship of three specific perinatal interventions – antenatal steroids, tocolysis and delivery by Caesarean section – to the chances of the baby being born in a good condition and to death in the delivery-room (DR). These outcomes may both be attributed directly to obstetric care, rather than the combined obstetric-neonatal input reflected in longer-term outcomes. We specifically sought to assess whether perinatal decision-making is solely responsible for improved short-term outcome, or whether there were additional, independent benefits conveyed by these obstetric interventions.

## Methods

Methods of case identification, data capture and other design aspects used in this study have been described previously [2]. All births in English hospitals between 22 and 26 competed weeks of gestation (i.e. 26 weeks and 6 days or less) occurring in 2006 to mothers normally resident in England were included. Data collection was in collaboration with the Centre for Maternal and Child Health Enquiries.

For the present study, the population was restricted to mothers with singleton pregnancies where the fetus was considered to be alive at admission to hospital and at either the start of monitoring of the labour or the point at which it was decided to perform Caesarean section. Terminations of pregnancy were excluded. Birth in a good condition was defined by the presence of a heart rate above 100 bpm at 5 min after birth, whereas “delivery-room death” includes all deaths during labour or in the delivery room.

The data were subject to a detailed exploratory analysis to investigate relationships between the different factors available, and also with the outcomes. In order to assess the individual effect of different exposures, it is necessary to examine each one separately, taking into consideration the effects of potential confounding variables as well as accounting for any random variation. Interpretation of results must then include the potential impact of any biases that may be present. Consequently, for this study, three factors were considered a priori as exposures: administration of antenatal steroids, use of tocolysis and Caesarean delivery.

### Study variables

Data items available to describe antenatal condition were: demographic data such as maternal age, ethnicity, body size and smoking status; maternal medical complications (diabetes either before or during pregnancy, hypertension or epilepsy); obstetric complications (prolonged premature rupture of membranes, abruption, antepartum haemorrhage after 20 weeks of gestation, pre-eclampsia or cervical incompetence warranting placement of a cervical suture); and fetal complications (intrauterine growth restriction and/or oligohydramnios).

Gestational age of the infant, determined by the earliest available ultrasound scan, was included as a categorical variable (per week) in the statistical analyses for ease of presentation. Fetal sex was considered in all analyses. Binary variables were created for labour type (spontaneous or none/induced), mode of delivery (vaginal or Caesarean) and presentation of the baby at delivery (cephalic or non-cephalic). The presence or suspicion of chorioamnionitis at any time was included as an antenatal risk factor, with maternal antibiotic administration prior to labour classified into treatment, prophylaxis or not prescribed. Administration of antenatal steroids was categorised into three levels – none, partial course (if a patient received the last dose less than 24 h prior to delivery), and full course (if the time interval to delivery was greater than one day). Tocolysis, although initially analysed by type of drug administered, was re-categorised in a binary fashion due to inadequate spread of data.

Provision of antenatal counselling was divided into the actual provision (Was there counselling by a senior obstetrician? Was there paediatric counselling?) and the content of the discussion. This included whether a decision to not perform Caesarean section in cases of fetal distress was made; whether or not withholding care was discussed; and whether the parents expressed any choice about resuscitation and provision of neonatal intensive care (provide full care for any live birth, withhold intensive care, or assess and provide care at paediatric discretion; or no choice expressed).

Health service factors were restricted to whether the mother had been transferred antenatally, and the level of neonatal care available at the delivering hospital. Maternal socio-economic status was based upon the Index of Multiple Deprivation 2007, using main residential postcode at the time of delivery. All data were collected contemporaneously with the birth onto paper forms and returned to a central office. Missing data were followed-up by contacting the reporting hospital until the data had been obtained, hence there are very few data missing. Ethical approval was granted by East London and City Research Ethics Committee (reference 05/Q0605/107) and permission was granted by the Patient Information Advisory Group (reference 3–07(f)/2005) to collect data without explicit consent, in conjunction with routine data collection performed for CEMACH (Confidential Enquiry into Maternal and Child Health) purposes.

### Statistical methods

Variables were categorised into fixed or background, pregnancy, obstetric clinical management, counselling and delivery related factors. Data within and between different groups were cross-tabulated to explore relationships; this aided the construction of diagrams of potential causal pathways. In turn, these facilitated identification of confounding variables and enabled selection of different sub-populations for sensitivity analyses. Each of the six exposure-outcome pairs was then investigated in a separate analysis, with remaining variables considered as potential confounders. All investigations were conducted using R [6].

For each analysis, the unadjusted odds ratio (OR) of the exposure on the outcome was calculated, followed by the OR for each potential confounding variable in association with both the exposure and the outcome. Univariable analysis was performed using the Mantel-Haenszel approach (or univariable logistic regression if the exposure had greater than one level) to determine which factors had an important effect. Evidence for effect modification by variables was assessed using a chi-squared test for homogeneity or likelihood ratio test (LRT) for each method, respectively; for this, a *p*-value of <0.1 was accepted. Next, multivariable analysis was employed using logistic regression with a forward step-wise approach, followed by assessment for interaction terms. A Wald test for association was used to assess the adjusted effect of individual factors on the outcome, with the LRT used to assess the relative importance between nested models. The significance level was set at *p* <0.05. Models were developed such that all biological/clinical variables were included prior to variables related to antenatal counselling.

The impact of missing data was accounted for in two ways. First, incomplete counselling data were recoded to show whether a response was recorded or not; the new variable was used to facilitate comparison with the whole data set. Secondly, we performed sensitivity analyses using two populations: for the steroid analyses, all patients for whom there was evidence of having received counselling, and for the tocolysis and mode of delivery analyses, the population that had received steroids.

## Results

### Population

**Fig. 1:**
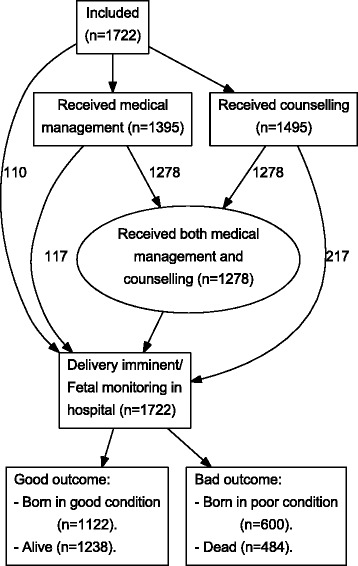
Principal pathways in the obstetric antecedents analysis. Flow diagram showing inclusion numbers, main pathways and principal outcomes for an assessment of the impact of obstetric interventions on condition at birth and delivery room death in the EPICure 2 cohort of babies born between 22 and 26 completed weeks gestation in England in 2006

In England during 2006, 2,466 singleton pregnancies were delivered between 22^+0^ and 26^+6^ weeks gestation; 532 fetal deaths occurred prior to maternal admission to hospital, 159 were alive at admission but died prior to the onset of monitoring during labour, and for 53 timing of death was unclear. Thus 1,722 women with a fetus known to be alive both at admission to hospital and at commencement of labour monitoring were included (Additional file 1: Figure S1). 1395 women received some form of medical treatment – including antibiotics, tocolysis, antenatal steroids and *in utero* transfer; of these, 1,278 were also recorded as having received counselling (Fig. 1), 1,213 women received steroids and 437 tocolysis, but only 406 women received both, meaning that there were 31 women who received tocolysis without receiving steroids as well (Additional file 2: Figure S3).

Data items were more than 95% complete for all but gestational age at booking (90.1%), maternal body mass index (86.0%), parity (45.0%) and counselling. Of the five items relating to counselling, 1,495 responses provided some indication that counselling had occurred, including 351 cases where a decision was made *not* to perform emergency Caesarean section in cases of fetal distress, 465 women with whom withholding care was discussed and 727 who expressed a choice regarding provision of resuscitation to and intensive care to a live born baby; additionally, 1,287 women were counselled by a senior obstetrician and 1,246 by a paediatrician (Additional file 1: Figure S2).

Four hundred and eighty-four (28.1%) babies died during labour or in the delivery room, 28 of whom were in a good condition at five minutes of age; overall, 600 (34.8%) babies had a heart rate below 100 beats per minute five minutes after delivery (Additional file 1: Figure S4). Each of the three exposures was associated with birth in good condition: unadjusted odds ratio (OR) for a partial course of antenatal steroids was 5.08 (95% confidence interval (CI): 3.84 – 6.72); for a full course 7.24 (95% CI: 5.60 – 9.36 with strong evidence of a trend (Chi-squared test for trend *p*<0.001); OR for tocolysis was 2.24 (95% CI: 1.75 – 2.90) and for delivery by Caesarean section 4.21 (95% CI: 3.06 – 5.80). Each exposure was also associated with lowered unadjusted odds of delivery-room death (partial course of steroids: 0.13, 95% CI: 0.10 – 0.18; full course: 0.09, 95% CI: 0.07 – 0.12; tocolysis: 0.33, 95% CI: 0.24 – 0.44; Caesarean delivery: 0.16, 95% CI: 0.10 – 0.24).

Univariate associations are shown in Additional file 2: Table S1. Both gestational age (in weeks) and male sex were strongly associated with condition at birth and delivery-room death. Both outcomes were associated with placental abruption, pre-eclampsia, *in utero* transfer, spontaneous labour, non-cephalic presentation and delivery in a centre with a level 3 neonatal intensive care unit. There were also strong associations between counselling variables and both outcomes.

In the population that had evidence of receiving counselling, the use of antenatal steroids was more frequent than in the overall population (Table 1). There was no difference in the use of tocolysis or surgical delivery, however, nor in fetal sex, gestational age profile or either of the outcomes. In contrast, steroid-receiving mothers were more frequently treated with tocolysis (33.47% compared with 25.41% in the overall population, *χ* ^2^ *p*-value <0.01) or delivered by Caesarean section (25.72% v. 20.73%, *p*<0.01), and there was a shift towards higher gestational age at delivery. There were no differences in terms of fetal sex or in the decision to not perform Caesarean section in the presence of fetal distress among steroid-treated mothers.

**Table 1:** Characteristics of key variables for the different study populations used in the obstetric antecedents investigation

Variable		“Complete” population		‘Counselled’ population			“Steroids only” population		
		N	%	N	%	*P*-value^a^	N	%	*P*-value^b^
Antenatal steroids		1701	—	1484	—	—	1213	—	—
	None	488	28.69	358	24.12	0.01	0	0.00	<0.01
	Partial	452	26.57	400	26.95		452	37.26	
	Full	761	44.74	726	48.92		761	62.74	
Tocolysis		1720	—	1495	—	—	1213	—	—
	No	1283	74.59	1085	72.58	0.21	807	66.53	<0.01
	Yes	437	25.41	410	27.42		406	33.47	
Delivery mode		1717	—	1495	—	—	1213	—	—
	Vaginal	1361	79.27	1170	78.26	0.51	901	74.28	<0.01
	Caesarean	356	20.73	325	21.74		312	25.72	
Gestational age		1722	—	1495	—	—	1213	—	—
	22 weeks	204	11.85	165	11.04	0.90	19	1.57	<0.01
	23 weeks	281	16.32	252	16.86		139	11.46	
	24 weeks	366	21.25	322	21.54		303	24.98	
	25 weeks	436	25.32	391	26.15		374	30.83	
	26 weeks	435	25.26	365	24.41		378	31.16	
Fetal sex		1719	—	1492	—	—	1213	—	—
	Female	804	46.77	699	46.85	0.99	586	48.31	0.43
	Male	915	53.23	793	53.15		627	51.69	
HR >100 5 min after birth		1722	—	1495	—	—	1213	—	—
	No	600	34.84	505	33.78	0.55	274	22.59	<0.01
	Yes	1122	65.16	990	66.22		939	77.41	
Delivery room death		1722	—	1495	—	—	1213	—	—
	No	1238	71.89	1078	72.11	0.92	1035	85.33	<0.01
	Yes	484	28.11	417	27.89		178	14.67	
Counselled by...		1722	—	1495	—	—	1213	—	—
	No one/not counselled	227	13.18	0	0.00	<0.01	87	7.17	<0.01
	Junior obstetrician only	168	9.76	168	11.24		94	7.75	
	Senior obstetrician only	165	9.58	165	11.04		96	7.91	
	Paediatrician and junior obstetrician	418	24.27	418	27.96		353	29.10	
	Paediatrician and senior obstetrician	744	43.21	744	49.77		583	48.06	
Decision to not perform C/S		1158	—	1017	—	—	820	—	—
	No	807	69.69	666	65.49	0.04	571	69.63	1.00
	Yes	351	30.31	351	34.51		249	30.37	
Resuscitation choice expressed		1235	—	1130	—	—	881	—	—
	No choice expressed	508	41.13	403	35.66	0.06	332	37.68	<0.01
	Full intensive care	391	31.66	391	34.60		346	39.27	
	Withhold intensive care	45	3.64	45	3.98		9	1.02	
	At paediatric discretion	291	23.56	291	25.75		194	22.02	
Discussion about withholding care		1178	—	1048	—	—	816	—	—
	Not discussed	713	60.53	583	55.63	0.02	545	66.79	0.01
	Discussed	465	39.47	465	44.37		271	33.21	

Characteristics of key variables from the EPICure 2 cohort study of births at 22–26 completed weeks of gestation in England in 2005. Mother-baby pairs were divided into three populations: the full population consisted of babies who were alive at admission to hospital and the commencement of labour monitoring or the decision to perform caesarean section (C/S). The two sub-populations studied in sensitivity analyses comprised mothers who received counselling, and those who received steroids

^a^Chi-squared test comparing distributions in the “counselled” and “full” populations

^b^Chi-squared test comparing distributions in the “steroids only” and “full” populations

### Antenatal steroids

Gestational age had the largest impact on the relationship between antenatal steroids and both outcomes. For condition at birth, gestational age reduced the ORs to 2.09 (95% CI: 1.51 – 2.90) and 2.53 (95% CI: 1.85 – 3.45) for partial and complete courses of steroids, respectively (Table 2) and, for delivery room death, the ORs were 0.39 (95% CI: 0.27 – 0.56) for partial and 0.35 (95% CI: 0.25 – 0.50) for a full course of steroids (Table 3). Gestational age was therefore included as the first potential clinical confounder in the multivariable analysis, followed by (in order) use of tocolysis, mode of delivery, NICU level at birth, *in utero* transfer, presence of spontaneous labour, and presentation at birth. Who the woman was counselled by, whether withholding care was discussed or the parents expressed a choice regarding resuscitation options, and the decision whether to perform Caesarean in case of fetal distress were included as potential counselling confounders. Additionally, there was evidence of effect modification by mode of delivery (LRT *p*<0.001 in the univariate analysis for condition at birth, *p*=0.008 for delivery room death) – but not from any other factors – hence this was entered into the model last.

**Table 2:** The effect of antenatal steroids on condition at birth

			Partial course		Full course		*P*-value^a^
		N	Effect	(95 % CI)	Effect	(95 % CI)	
Confounding only models							
	Baseline	1701	5.08	(3.84 – 6.72)	7.24	(5.60 – 9.36)	—
	Baseline + GA	1701	2.09	(1.51 – 2.90)	2.53	(1.85 – 3.45)	<0.001
	’Clinical’ model^b^	1602	1.42	(0.99 – 2.05)	1.64	(1.14 – 2.36)	0.013
	Complete model^c^	1602	1.38	(0.93 – 2.03)	1.82	(1.22 – 2.71)	<0.001
Models with effect modification by mode of delivery^b, c^							
	Clinical model: Vaginal delivery	1602	1.61	(1.09 – 2.37)	1.66	(1.13 – 2.44)	0.018
	Clinical model: Caesarean delivery		1.31	(0.46 – 3.69)	0.48	(0.17 – 1.39)	
	Complete model: vaginal delivery		1.84	(1.20 – 2.82)	1.63	(1.08 – 2.47)	0.021
	Complete model: Caesarean delivery		1.23	(0.42 – 3.61)	0.42	(0.14 – 1.26)	

Odds ratios of the effect of antenatal steroids on the presence of a heart rate greater than 100 at minutes of age in the complete population of babies born at 22–26 completed weeks of gestation in England in 2006 who were known to be alive at admission to hospital and at the start of labour monitoring or delivery to perform Caesarean section

^a^Likelihood ratio test *p*-value, comparing current to next simplest model described

^b^The clinical model for condition at birth was adjusted for: gestational age, tocolysis, mode of delivery, NICU level at birth, *in utero* transfer, presentation at delivery

^c^The complete models were adjusted for the same factors as the clinical models, plus: who the parents were counselled by, what choice was expressed for resuscitation, and whether a decision was made to not perform Caesarean section in the presence of fetal distress

**Table 3:** The effect of antenatal steroids on delivery room death

			Partial course		Full course		*P*-value^a^
		N	Effect	(95% CI)	Effect	(95% CI)	
Confounding only models							
	Baseline	1701	0.13	(0.10 – 0.18)	0.09	(0.07 – 0.12)	—
	Baseline + GA	1701	0.39	(0.27 – 0.56)	0.35	(0.25 – 0.50)	<0.001
	’Clinical’ model^b^	1656	0.47	(0.32 – 0.70)	0.45	(0.30 – 0.67)	0.215
	Complete model^c^	1656	0.47	(0.31 – 0.74)	0.37	0.23 – 0.58)	<0.001
Models with effect modification by mode of delivery^b, c^							
	Clinical model: vaginal delivery	1656	0.44	(0.29 – 0.67)	0.42	(0.28 – 0.63)	0.10
	Clinical model: caesarean delivery		0.72	(0.21 – 2.50)	1.63	(0.44 – 6.09)	
	Complete model: vaginal delivery		0.34	(0.21 – 0.55)	0.41	(0.26 – 0.64)	0.083
	Complete model: caesarean delivery		0.93	(0.24 – 3.63)	2.06	(0.49 – 8.65)	

Odds ratios of the effect of antenatal steroids on delivery room death in the complete population of babies born at 22–26 completed weeks of gestation in England in 2006 who were known to be alive at admission to hospital and at the start of labour monitoring or delivery to perform Caesarean section

^a^Likelihood ratio test *p*-value, comparing current to next simplest model described

^b^The clinical model for delivery room death was adjusted for: gestational age, tocolysis, mode of delivery, NICU level at birth, *in utero* transfer, and presence of a spontaneous labour

^c^The complete models were adjusted for the same factors as the clinical models, plus: who the parents were counselled by, what choice was expressed for resuscitation, and whether a decision was made to not perform Caesarean section in the presence of fetal distress

All clinical confounders except spontaneous/induced labour contributed to the final model for birth in good condition (adjusted ORs (aOR) for a partial course 1.42 (95% CI: 0.99 – 2.04); complete course: 1.64 (95% CI: 1.16 – 2.35). The only counselling variable not affecting the final model related to whether or not withholding care was discussed. Even after the other counselling variables were included, a complete course of steroids retained a strong effect (aOR of 1.82 (95% CI: 1.22 – 2.71)). Almost the same factors were influential on the odds of delivery room death, except for delivery presentation which had no effect and presence of a spontaneous labour which did. Results adjusted for clinical variables show a change in the odds ratio to 0.47 (95% CI: 0.32 – 0.70) for a partial course and 0.45 (95% CI: 0.30 – 0.67) for a full course of steroids. After adjustment for counselling factors, the ORs were 0.47 (95% CI: 0.31 – 0.73) and 0.37 (95% CI: 0.23 - 0.58) for partial and full courses, respectively.

When taking into account the potential interaction with mode of delivery, there was no evidence of birth in an improved condition after administration of steroids antenatally for those babies born by Caesarean section, but a strong benefit to babies born vaginally (LRT *p*=0.018). Those who received a partial course of steroids demonstrated an improved odds ratio of 1.61 (95% CI: 1.09 - 2.37) which increased further to 1.84 (95% CI: 1.20 – 2.82) after the inclusion of counselling variables, as shown in Table 2. In contrast, the OR for those born after a complete course of steroids decreased slightly from 1.66 (95% CI: 1.13 – 2.44) to 1.63 (95% CI: 1.08 – 2.47). These findings were reflected for delivery room death; full results are in Table 3.

#### Sensitivity analysis

We repeated the analyses in the population who received counselling. Potential confounders were the same as for the primary analysis except the presence or absence of labour had no effect when condition at birth was the outcome, and level of neonatal unit at birth had no effect of steroids on either outcome. Gestational age remained the biggest confounding factor, with no evidence of differing effects by gestational age (LRT *p*=0.44 for condition at birth and *p*=0.46 for delivery room death). As for the primary analysis, there was evidence of interaction with mode of delivery, with no effect seen in those born by Caesarean section, but marked effects seen in babies born vaginally for both partial and full courses of steroids; full results are presented in Additional file 2: Table S2.

### Tocolysis

Women who were treated with tocolytics (*n*=437) during the study period predominantly received atosiban (*n*=143, 32.7%) or nifedipine (*n*=189, 43.3%). Other drugs were given to 53 women (12.1%), 40 (9.2%) received multiple drugs, and 12 women (2.7%) were recorded as having tocolysis without the type of tocolytic being recorded. As the study was not powered to examine individual tocolytic drug effects, subjects were re-classified as either having received tocolysis or not. Overall, tocolysis was associated with a more frequent good outcome (unadjusted OR 2.25, 95% CI: 1.75 – 2.90).

Univariate analysis identified gestational age, antenatal steroids, neonatal care level available at birth, *in utero* transfer, labour type and placental abruption as potential clinical confounders of the relationships with both outcomes. Of the listed factors, all except labour type had an important confounding effect between the use of tocolysis and condition at birth, reducing the odds ratios to 1.37 (95% CI: 1.01 – 1.87). After adding in variables representing who provided counselling (if any was provided), parental choice regarding neonatal resuscitation, and decision to not perform Caesarean section for fetal distress, the odds ratio was 1.45 (95% CI: 1.05 – 2.00). The inclusion of whether or not withholding care was discussed did not affect the model, nor was there any evidence of effect modification by any variable. The findings for delivery room death were very similar, with the final model including identical variables and demonstrating an effect by tocolysis of 0.48 (95% CI: 0.32 – 0.73). Results are shown in Table 4.

**Table 4:** Tocolysis regression results

Model		Complete population				Steroids-only population			
		N	OR	95% CI	*P*-value^a^	N	OR	95% CI	*P*-value^a^
Condition at birth									
	Baseline	1720	2.25	(1.75 – 2.90)	<0.0001	1213	1.44	(1.08 – 1.95)	0.0138
	Baseline + GA^b^	1720	1.64	(1.24 – 2.18)	<0.0001	1213	1.53	(1.12 – 2.09)	<0.0001
	Clinical^c^	1655	1.37	(1.01 – 1.87)	<0.0001	1205	1.42	(1.04 – 1.96)	<0.0001
	Counselling^d^	1655	1.45	(1.05 – 2.00)	<0.0001	1205	1.47	(1.06 – 2.04)	<0.0001
Delivery Room death									
	Baseline	1720	0.33	(0.24 – 0.44)	<0.0001	1213	0.53	(0.36 – 0.76)	0.0005
	Baseline + GA^b^	1720	0.46	(0.32 – 0.65)	<0.0001	1213	0.47	(0.31 – 0.70)	<0.0001
	Clinical^c^	1655	0.58	(0.39 – 0.84)	<0.0001	1211	0.49	(0.32 – 0.73)	<0.0001
	Counselling^d^	1655	0.48	(0.32 – 0.73)	<0.0001	1211	0.43	(0.28 – 0.66)	<0.0001

Odds ratios of the effect of tocolysis on the presence of a heart rate greater than 100 at minutes of age or to delivery room death in the complete population of babies born at 22–26 completed weeks of gestation in England in 2006 who were known to be alive at admission to hospital and at the start of labour monitoring or delivery to perform Caesarean section, and the restricted population of those babies born to women who received antenatal steroids

^a^Likelihood ratio test *p*-value comparing model against next simplest model

^b^GA: gestational age

^c^The clinical models for condition at birth and delivery room death were adjusted for: provision of antenatal steroids, NICU level at birth, *in utero* transfer and presence of placental abruption

^d^In addition to the factors adjusted for in the clinical models, the complete models were also adjusted for who counselling was provided by, whether and what choice was expressed for resuscitation, and whether a decision was made to *not* perform Caesarean section in the presence of fetal distress

#### Sensitivity analysis

The effect of tocolysis was also studied in the population of babies whose mothers received steroids. The same factors plus the presence of maternal pre-eclampsia were considered as potential confounders. Neither partial nor full courses of antenatal steroids, *in utero* transfer or labour type modified the effect of tocolysis on condition at birth, with the clinical model showing an odds ratio of 1.42 (95% CI: 1.04 – 1.96) and the complete model an OR of 1.47 (95% CI: 1.06 – 2.04). For delivery room death, gestational age and placental abruption were the only clinical factors identified as playing an important confounding role, leading to an adjusted OR of 0.49 (95% CI: 0.32 – 0.73). Of the counselling factors, all except whether withholding care was discussed made an important contribution to the final model (OR 0.44 (95% CI: 0.28 – 0.67)); there was no evidence of interaction. Results are tabulated alongside the complete population in Table 4.

### Mode of delivery

Gestational age was the strongest factor confounding the relationship between Caesarean section and both outcomes, changing the OR for condition at birth from 4.21 (95% CI: 3.06 – 5.80) to 1.63 (95% CI: 1.15 – 2.33), and for delivery room death from 0.16 (95% CI: 0.11 – 0.24) to 0.67 (95% CI: 0.42 – 1.05). However, there was important evidence of effect modification (likelihood ratio test *p*=0.017 for the relationship with condition at birth and *p*=0.016 for that with delivery room death) by week of gestational age. Because very few Caesarean sections were performed at low gestational ages (1, 6 and 42 at 22, 23 and 24 weeks gestation, respectively), we collapsed the 22–24 week categories into a single group. Caesarean delivery was then associated with birth in a good condition at ≤24 and 25 weeks (ORs 3.89 (95% CI: 2.00 – 7.58) and 1.85 (95% CI: 1.02 – 3.34)), but not at 26 weeks gestation (0R 1.22, 95% CI: 0.69 – 2.18). For delivery room death, a difference was only shown for those born at 24 weeks or below (0.24, 95% CI: 0.12 – 0.50), with no association at either 25 or 26 weeks (0.79, 95% CI: 0.39 – 1.63, and 0.63, 95% CI: 0.24 – 1.70, respectively).

A further consideration is the presence or not of labour: only 64 women underwent Caesarean section following spontaneous onset of labour, compared with 292 who were not in labour. Including this removed the association with condition at birth for women in spontaneous labour, but demonstrated a strong association in women who were not in labour at both 24 weeks and below (OR 13.5, 95% CI: 3.50 – 52.08), and 25 weeks (4.13, 95% CI: 1.35 – 12.62). These findings were little changed after the addition of the remaining clinical variables (Table 5) There were similar findings for delivery room death, with no evidence of an effect from Caesarean delivery after spontaneous labour and marked evidence for babies born to women not in labour at ≤24 and 25 weeks, but no effect for those born at 26 weeks (Table 5).

**Table 5:** Delivery regression results

Model		N	≤24 weeks		25 weeks		26 weeks		*P*-value^a^
			OR	(95% CI)	OR	(95% CI)	OR	(95% CI)	
Condition at birth									
	Baseline	1717	3.89	(2.00 – 7.58)	1.85	(1.02 – 3.34)	1.22	(0.69 – 2.18)	0.031
	Baseline: no labour	1712	13.5	(3.50 – 52.08)	4.13	(1.35 – 12.62)	0.98	(0.11 – 8.41)	0.018
	Baseline: spontaneous labour		1.79	(0.76 – 4.24)	1.95	(0.79 – 4.79)	0.75	(0.35 – 1.58)	
	Clinical: no labour^b^	1680	22.96	(5.19 – 101.52)	8.24	(2.49 – 27.33)	2.22	(0.24 – 20.16)	0.001
	Clinical: spontaneous labour^b^		1.18	(0.47 – 2.96)	2.01	(0.80 – 5.08)	0.71	(0.33 – 1.54)	
	Complete: no labour^c^	1680	12.67	(2.79 – 57.60)	4.94	(1.44 – 16.90)	1.56	(0.16 – 14.81)	<0.001
	Complete: spontaneous labour^c^		0.95	(0.38 – 2.40)	1.58	(0.62 – 4.03)	0.70	(0.32 – 1.52)	
Delivery room death									
	Baseline	1717	0.24	(0.12 – 0.50)	0.79	(0.39 – 1.63)	0.63	(0.24 – 1.70)	0.055
	Baseline: no labour	1712	0.05	(0.01 – 0.23)	0.20	(0.06 – 0.67)	0.39	(0.04 – 3.76)	0.019
	Baseline: spontaneous labour		0.56	(0.23 – 1.35)	0.82	(0.27 – 2.44)	0.65	(0.14 – 2.97)	
	Clinical: no labour^b^	1680	0.01	(0.00 – 0.09)	0.06	(0.02 – 0.25)	0.09	(0.01 – 1.03)	<0.001
	Clinical: spontaneous labour^b^		0.96	(0.36 – 2.55)	0.77	(0.25 – 2.43)	0.63	(0.13 – 3.00)	
	Complete: no labour^c^	1680	0.03	(0.01 – 0.21)	0.13	(0.03 – 0.55)	0.12	(0.01 – 1.63)	<0.001
	Complete: spontaneous labour^c^		1.36	(0.50 – 3.70)	1.07	(0.33 – 3.46)	0.63	(0.13 – 3.00)	

Odds ratios of the effect of delivery by caesaerean section on the presence of a heart rate greater than 100 at minutes of age or to delivery room death in the complete population of babies born at 22–26 completed weeks of gestation in England in 2006 who were known to be alive at admission to hospital and at the start of labour monitoring or delivery to perform Caesarean section

^a^Likelihood ratio test comparing model against next simplest model

^b^The clinical models were additionally adjusted for: antenatal steroids, *in utero* transfer, maternal pre-eclampsia and placental abruption

^c^The complete models were adjusted for the same factors as the clinical models, plus: who the parents were counselled by, whether a decision was made not to perform Caesarean section in cases of fetal distress, what choice was expressed for resuscitation, and whether withholding care was discussed

#### Sensitivity analysis

In the steroids-only population, there was strong evidence for both outcomes of effect modification by labour type, but gestational age only acted as a confounder. For condition at birth as the outcome, in utero transfer, presentation at delivery, the level of neonatal care available at the birth hospital, maternal pre-eclampsia and placental abruption were also identified as confounders, leading to adjusted ORs of 8.67 (95% CI: 3.47 – 21.70) and 1.22 (95% CI: 0.71 – 2.09) for women who were not in labour and those in spontaneous labour, respectively. In the model including counselling, who the subject was counselled by, whether a decision was made not to perform Caesarean section in the presence of fetal distress, and whether or not a choice was expressed about resuscitation all had important effects, resulting in an OR for women not in labour of 5.55 (95% CI: 2.13 – 14.47) whereas for women in labour there was no evidence of an effect (Additional file 2: Table S3).

In addition to gestational age and labour type, confounders of the effect of Caesarean delivery on delivery room death were the length of course of antenatal steroids (partial or full), in utero transfer, presentation at delivery, maternal pre-eclampsia and placental abruption. Inclusion of these reduced the odds of dying for babies born to women not in labour to 0.05 (95% CI: 0.02 – 0.17), with no effect in women who were in spontaneous labour (OR 0.90, 95% CI: 0.45 – 1.81). There was little change following the inclusion of all counselling variable (Additional file 2: Table S3).

## Discussion

### Principal findings

This study produced strong evidence that antenatal steroids are associated with improved survival at birth as well as with being born in better condition for singletons born at 22 to 26+6 weeks gestation. These results were obtained even after accounting for potential decision-making influences, and particularly in those babies born vaginally. Tocolysis was also associated with improvements in both outcomes. In contrast, mode of delivery was subject to effect modification by gestational age and by the presence or not of spontaneous labour. There was no evidence of an effect by Caesarean section at any gestation in women with spontaneous labour, but in women without spontaneous labour there were bigger effects at lower gestations. This finding was even more pronounced for the outcome of delivery room death than for condition at birth.

### Strengths and limitations of this study

Our study benefits from the size and the completeness of the data that were available: it covers the entire population of England, and data collection was comprehensive [2]. It was therefore possible to obtain robust statistical evidence in support of results. This provides a high degree of certainty about how the usage of antenatal steroids, tocolysis and operative delivery affected immediate outcomes for these extremely premature babies in 2006 in England. However, many of the regression analyses were complex, particularly when considering interaction, resulting in small numbers within individual cells and an increased possibility of error. This is reflected by wide confidence intervals in results – particularly evident in the mode of delivery analyses.

Non-reporting, selection bias may have arisen due to small numbers of missing data points. However, this would tend to weaken the effects reported. For example, missing data were most common in the gestation at which mother booked for care; no differences were seen in reported data comparing late versus early booking, however, it is equally plausible that there was no effect anyway.

More importantly, the counselling variables were incompletely filled out. We therefore based analyses on whether there was evidence that counselling was carried out or not. This may have led to us underestimating the confounding impact of these variables if there was under-recording of positive responses; we think it is more likely that positive responses will have been recorded, and that in those subjects where a response was missing the relevant conversation is less likely to have occurred. The incomplete counselling variables may particularly have affected the sensitivity analyses for the effect of antenatal steroids. In actual fact, the point estimates obtained from the sensitivity analyses are more biologically plausible than the main analyses as a dose-response relationship is seen in all models. Reassuringly, effect magnitudes and confidence intervals are similar across both sets of analyses (using the main and counselled populations).

Recall bias was minimised as data were collected contemporaneously, but there may have been differential reporting at the various study centres leading to differential misclassification. Clear instructions were provided with the initial data collection form in order to prevent this, and the data are internally consistent [2]. Thus, this is likely to have had little if any impact on the results. Within the statistical analyses, a conservative attitude was taken in interpreting ambiguous data; this meant that interpretation was restricted on occasion. For example, chorioamnionitis is a diverse condition that is poorly diagnosed on both clinical and histological grounds [7]. Because of the wording of the questions that had been used, the analysis was only able to look at the combined group of “clinical suspicion of chorioamnionitis at any time before birth” and those in whom “chorioamnionitis [was] noted at time of birth”, rather than more precise patient categories. Any misclassification that resulted from this would have happened in a non-differential fashion, and therefore be likely to bias results towards the null.

Some may argue our choices of confounding variables in the analyses are wrong; particularly, in the steroids analysis, the inclusion of mode of delivery as a covariate may appear bizarre, as it seems to be on the causal pathway, as shown when considering Additional file 1: Figures S3 and S4 together. However, mode of delivery may instead be considered as a marker for *intention* to deliver – for, having made a decision to delivery operatively (e.g. in the presence of intrauterine growth retardation), one would first ensure that steroids had been administered; in this scenario, the model then makes sense.

Our results may, however, be impacted by residual confounding. More detail could have been obtained about the current variables: for instance, we have little detail regarding tocolysis – how long women were administered drugs for, or at which doses. However, we believe we have captured the most important variables: this is demonstrated through confounding by gestational age and the use of antenatal steroids, which had consistently large impacts throughout all analyses. Furthermore, a longer study period or a larger baseline population would be required to power more detailed investigations.

More specifically, residual confounding may have occurred in relation to the counselling information. The low completion rates for these variables in comparison to other questions, together with the patterns of overlap in the responses, suggests there was some confusion in answering these questions. This raises the possibility that the true confounding effects of perinatal decision-making at the borders of viability have not been accounted for. However, when analyses excluding counselling variables (“clinical” models in Tables 2, 3, 4 and 5 and Additional file 2: Tables S2 and S3) are compared with those that include them (the “complete” models), there tended to be a *more* pronounced effect in the complete (i.e. including counselling factors) models. We also find reassurance in our results by recognising that effects are in the direction and of a magnitude that might be expected.

### Study findings in context

There are very marked improvements in survival and condition at birth in babies born via operative delivery at low gestations to women who were not in labour, while no benefit is seen from performing Caesarean sections for women who are already in labour. This suggests that there is an important influence from case selection: very few Caesarean sections were performed at the lowest gestations (1, 6 and 42 at 22, 23 and 24 weeks respectively) where the odds of a favourable outcome are the highest. At these gestations, Caesarean section will only be offered to women who are not in labour if the fetus is considered viable, thus making these findings difficult to interpret.

Our findings are compatible with a Cochrane Review investigating the effect of Caesarean delivery in premature singleton babies that found no evidence of a difference in mortality [8]. However, the review identified only six studies, three of which examined mortality, and only two of these including births as low as 26 weeks gestational age [8]. Our results are also compatible with another study using registry data from the United States [9]. That study used a “trimming procedure” to ensure that only births with congruent birth weight and gestational age data were included. Neonatal mortality was lower in babies born operatively at 25 weeks and below, with more pronounced differences with decreasing gestational age [9].

Tocolysis was associated with an improved outcome in all groups, even after accounting for antenatal steroids, gestational age, perinatal counselling and other factors. These data therefore suggest that therapy is appropriately targeted by current obstetric practice within the population of women who present with threatened extremely preterm labour. There are a plethora of studies and systematic reviews relating to specific questions about tocolysis – for instance, a recent network meta-analysis identified 95 randomised controlled trials comparing tocolytics with either each other or placebo, [10] and observed that no single tocolytic drug was associated with improved neonatal mortality or decreased morbidity. However, there were no data presented on the effect of gestational age in any of the analyses [10] and very few data evaluate the impact of tocolysis on neonatal mortality at extremely low gestational ages [11, 12].

Perhaps most notably, in independent analysis, antenatal steroids were consistently associated with improved outcomes; they were also among the strongest confounders for the other exposures. This is worth highlighting as, while the results are similar to other studies that have looked at longer term survival [13–16] or shorter term outcomes in the population of babies admitted into neonatal intensive care, [16] there is little biological reason apparent for steroids to cause an improvement in condition at birth or survival in the delivery room.

An alternative possibility is that steroids may simply be a marker of other reasons for the improved survival rate. This fact would warrant further investigation if we had not adjusted for prior intention (by both parents and clinical teams) through the use of data related to perinatal counselling. Despite our questions being crude, we are confident that they captured an important aspect of the management as, with the exception of whether withholding care was discussed or not, all counselling variables made important contributions towards the final models in all analyses. While a future study could investigate this further by including more – and more detailed – questions specifically related to the intention to resuscitate and provision of further care, difficulty remains in ensuring adequate response rates. Consequently, if our findings are true, there is a biological effect from the administration of antenatal steroids that improves short-term survival – an effect that is particularly marked in those born by vaginal delivery. This warrants further study.

## Conclusion

In conclusion, we have demonstrated the effect of antenatal steroids, tocolysis and mode of delivery on the chances of being born in a good condition and of delivery-room death for babies delivered at 26 completed weeks gestational age or below in England using data from a 2006 birth cohort. The results demonstrate the importance of antenatal steroids in determining early condition of babies born vaginally, and provide estimates of the impact of tocolysis and Caesarean section that may be used when counselling patients and their families.

## Additional files

Additional file 1 Supplementary Figures. Supplementary Figure 1 — Inclusion numbers for the obstetric antecedents analysis Supplementary Figure 2 — Counselling factors assessed in the obstetric antecedents analysis Supplementary Figure 3 — Clinical factors assessed in the obstetric antecedents analysis Supplementary Figure 4 — Delivery information available for the obstetric antecedents analysis. (PDF 47 KB)

Additional file 2 Supplementary Tables. Supplementary Table 1 — Descriptive statistics from the obstetric antecedents investigation Supplementary Table 2 — Steroids sensitivity analysis results Supplementary Table 3 — Sensitivity analysis results for mode of delivery. (PDF 95 KB)

Additional file 3 STROBE Statement checklist. (PDF 67 KB)

## Abbreviations

aOR: Adjusted odds ratio
bpm: Beats per minute
CEMACH: Confidential Enquiry into Maternal and Child Health
CI: Confidence interval
DR: Delivery room
LRT: Likelihood ratio test
NICU: Neonatal intensive care unit
OR: Odds ratio
