# Supplementary material for: Impact of obstetric interventions on condition at birth in extremely preterm babies: evidence from a national cohort study
Source: BMC Pregnancy Childbirth. 2016 Dec 13;16:390. doi: 10.1186/s12884-016-1154-y (PMC5154160; doi:10.1186/s12884-016-1154-y)
Supplement: Additional file 1 — Supplementary Figures. Supplementary Figure 1 — Inclusion numbers for the obstetric antecedents analysis Supplementary Figure 2 — Counselling factors assessed in the obstetric antecedents analysis Supplementary Figure 3 — Clinical factors assessed in the obstetric antecedents analysis Supplementary Figure 4 — Delivery information available for the obstetric antecedents analysis. (PDF 47 KB) [file 12884_2016_1154_MOESM1_ESM.pdf]

RESEARCH

Additional file 1: Supplementary figures

Andrei S Morgan[1*], Neil Marlow[1], Elizabeth S Draper[3], Zarko Alfirević[4], Enid M Hennessy[5] and Kate Costeloe[2]

*Correspondence:
andrei.morgan@ucl.ac.uk
[1]Institute for Womens' Health, UCL, 74 Huntley Street, WC1E 6AU, London, UK
Full list of author information is available at the end of the article

Supplementary figure 1 — Inclusion numbers for the obstetric antecedents analysis

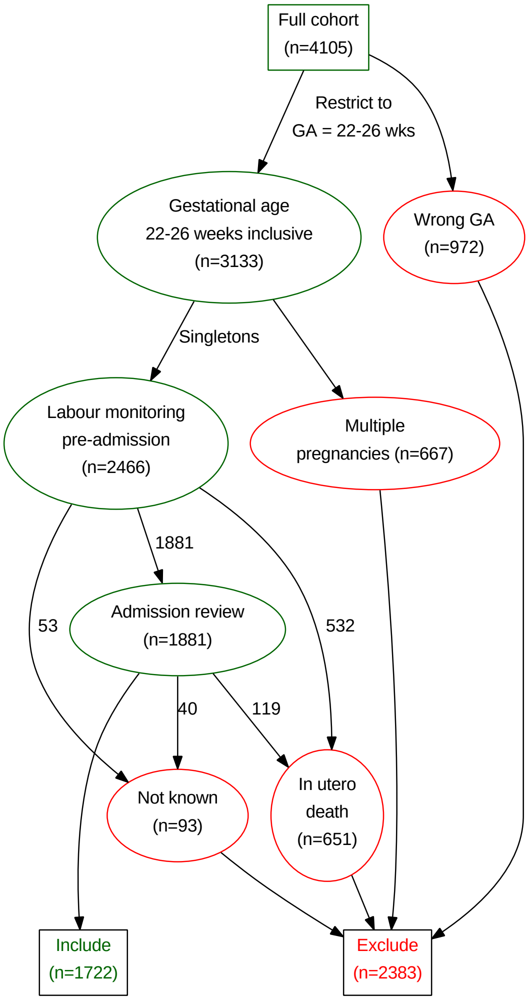

Flow diagram showing inclusion and exclusion numbers for participant mother-baby pairs from the EPICure 2 cohort of babies born between 22 and 26 completed weeks gestation in England in 2006. GA: gestational age.

Supplementary figure 2 — Counselling factors assessed in the obstetric antecedents analysis

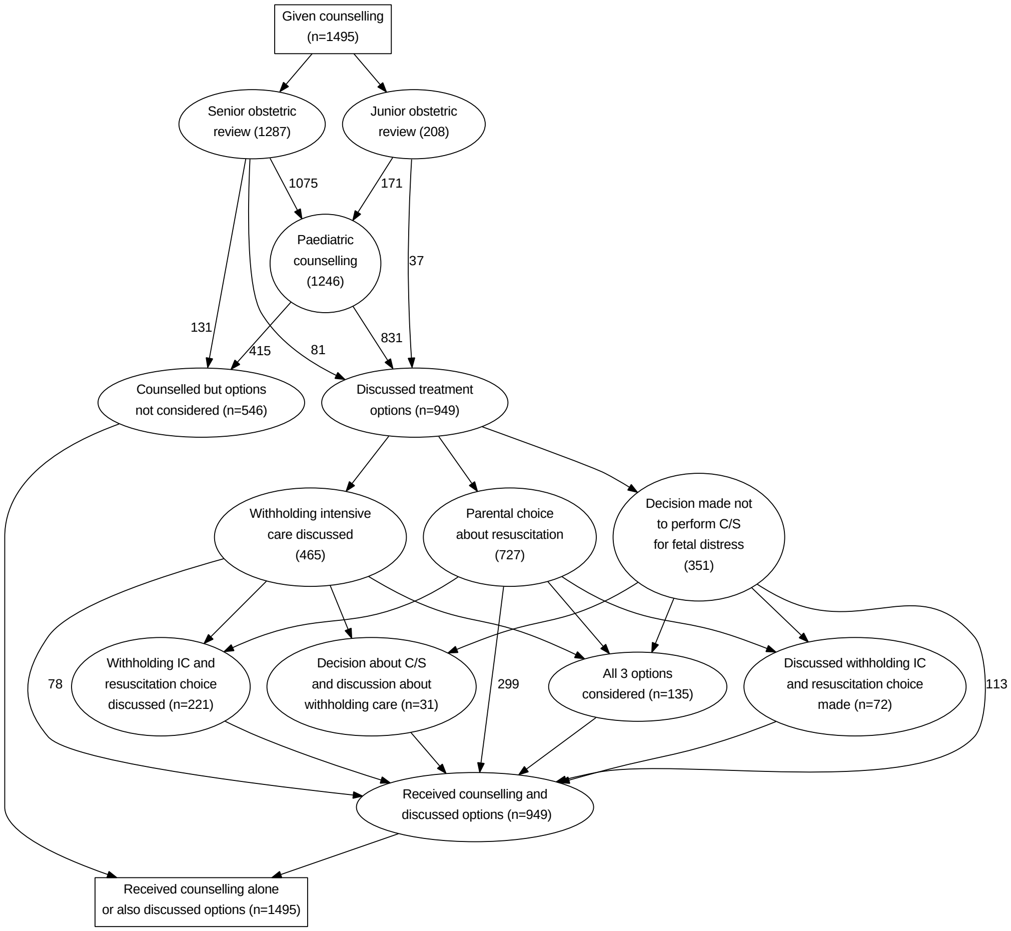

Flow diagram showing detail about counselling factors measured in the EPICure 2 cohort of babies born between 22 and 26 completed weeks gestation in England in 2006.

Supplementary figure 3 — Clinical factors assessed in the obstetric antecedents analysis

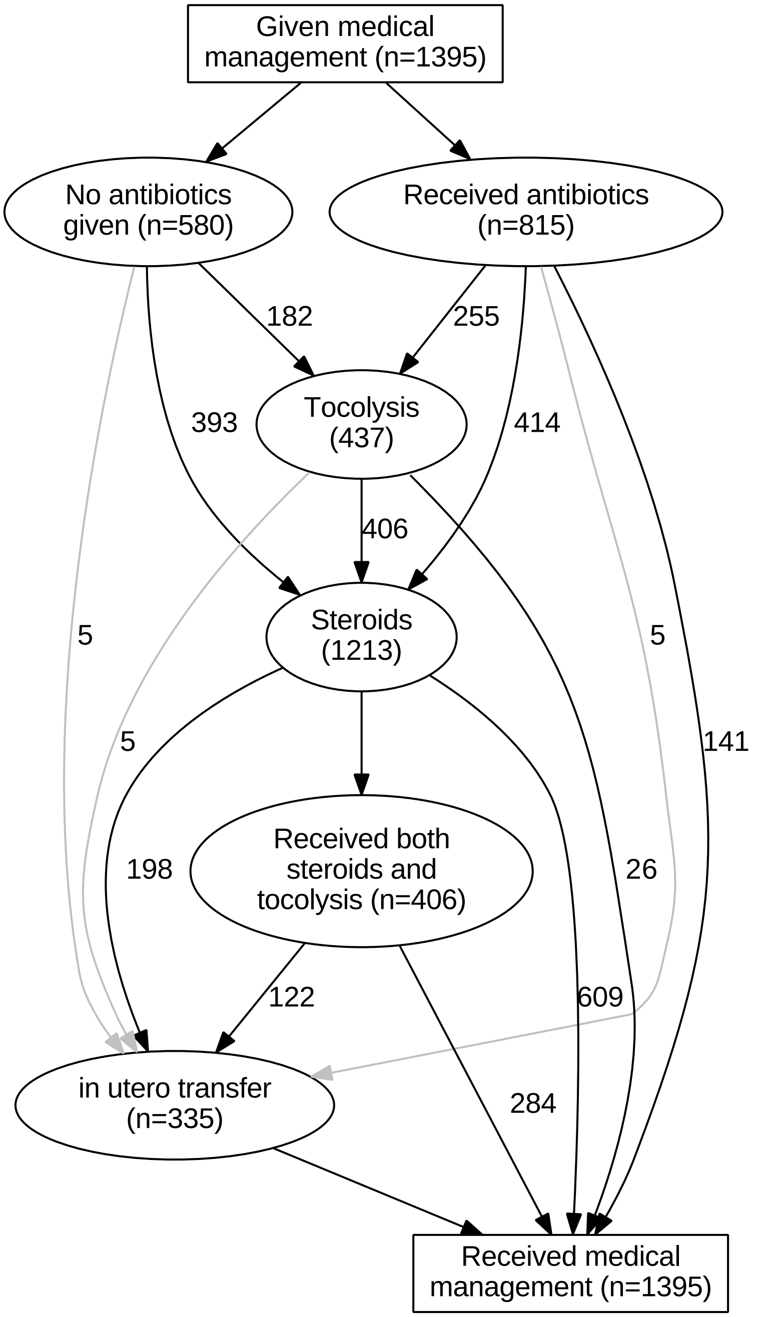

Flow diagram showing detail about clinical factors measured in the EPICure 2 cohort of babies born between 22 and 26 completed weeks gestation in England in 2006.

Supplementary figure 4 — Delivery information available for the obstetric antecedents analysis

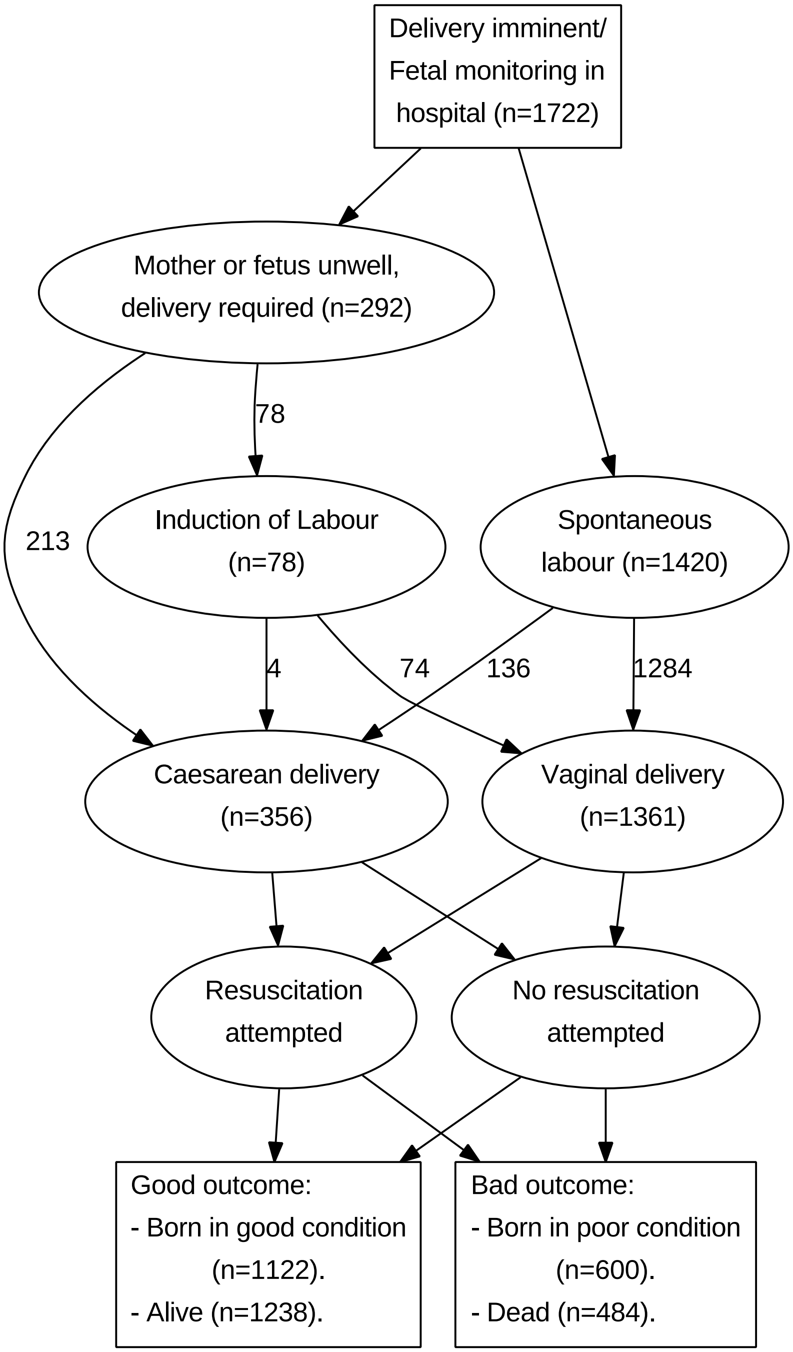

Flow diagram showing detail about delivery measured in the EPICure 2 cohort of babies born between 22 and 26 completed weeks gestation in England in 2006. Not all numbers add to 1,722 due to missing data in some of the variables.

Author details
[1]Institute for Womens' Health, UCL, 74 Huntley Street, WC1E 6AU, London, UK. [2] Homerton Hospital, London, UK. [3] University of Leicester, Leicester, UK. [4] University of Liverpool, Liverool, UK. [5] The Wolfson Institute, Queen Mary University of London, London, UK.

References
