# Supplementary material for: Impact of obstetric interventions on condition at birth in extremely preterm babies: evidence from a national cohort study
Source: BMC Pregnancy Childbirth. 2016 Dec 13;16:390. doi: 10.1186/s12884-016-1154-y (PMC5154160; doi:10.1186/s12884-016-1154-y)
Supplement: Additional file 2 — Supplementary Tables. Supplementary Table 1 — Descriptive statistics from the obstetric antecedents investigation Supplementary Table 2 — Steroids sensitivity analysis results Supplementary Table 3 — Sensitivity analysis results for mode of delivery. (PDF 95 KB) [file 12884_2016_1154_MOESM2_ESM.pdf]

RESEARCH

Additional file 2: Supplementary tables

Andrei S Morgan[1*], Neil Marlow[1], Elizabeth S Draper[3], Zarko Alfirević[4], Enid M Hennessy[5] and Kate Costeloe[2]

*Correspondence: andrei.morgan@ucl.ac.uk
[1]Institute for Womens' Health, UCL, 74 Huntley Street, WC1E 6AU, London, UK
Full list of author information is available at the end of the article

Supplementary table 1 — Descriptive statistics from the obstetric antecedents investigation

This table is shown on the following pages in landscape format.

Table S1: Descriptive statistics of explanatory variables in relation to presence of a heart rate greater than 100 at five minutes of age (“good condition”) or to death during labour or in the delivery room (“delivery room death”) in the complete population of babies born at 22-26 completed weeks of gestation in England in 2006 who were known to be alive at admission to hospital and at the start of labour monitoring or decision to perform Caesarean section.

**Variable (Levels)**	**N**	**(%)**	**Born in good condition**	**(HR %)**	**OR**	**(95% CI)**	**P-value**	**Peri-natal death**	**(%)**	**OR**	**(95% CI)**	**P-value**
Antenatal steroid course[a] *N=1701*												
none	488	28.3	172	35.2	1.00	(—)	—	301	61.7	1.00	—	(—)
partial	452	26.2	332	73.5	5.08	(3.84 – 6.72)	< 0.001	79	17.5	0.13	(0.10 – 0.18)	< 0.001
full	761	44.2	607	79.8	7.24	(5.60 – 9.36)	< 0.001	99	13.0	0.09	(0.07 – 0.12)	< 0.001
Tocolysis *N=1720*												
no	1283	74.5	782	61.0	1.00	(—)	—	423	33.0	1.00	(—)	—
yes	437	25.4	340	77.8	2.25	(1.75 – 2.89)	< 0.001	61	14.0	0.33	(0.25 – 0.44)	< 0.001
Delivery by Caesarean *N=1717*												
no	1361	79.0	814	59.8	1.00	—	(—)	456	33.5	1.00	—	(—)
yes	356	20.7	307	86.2	4.21	(3.06 – 5.80)	< 0.001	27	7.6	0.16	(0.11 – 0.24)	< 0.001
Gestational age (weeks) *N=1722*												
22	204	11.8	22	10.8	0.02	(0.01 – 0.03)	< 0.001	190	93.1	297.14	(145.89 – 605.20)	< 0.001
23	281	16.3	127	45.2	0.12	(0.09 – 0.18)	< 0.001	141	50.2	22.05	(13.16 – 36.94)	< 0.001
24	366	21.3	243	66.4	0.30	(0.21 – 0.42)	< 0.001	89	24.3	7.03	(4.19 – 11.81)	< 0.001
25	436	25.3	352	80.7	0.63	(0.44 – 0.91)	0.02	45	10.3	2.52	(1.45 – 4.38)	< 0.001
26	435	25.3	378	86.9	1.00	(—)	—	19	4.4	1.00	(—)	—
Fetal sex *N=1719*												
Female	804	46.7	549	68.3	1.00	(—)	—	201	25.0	1.00	(—)	—
Male	915	53.1	572	62.5	0.77	(0.63 – 0.95)	0.01	280	30.6	1.32	(1.07 – 1.64)	0.01
Ethnicity *N=1722*												
White	1118	64.9	725	64.8	1.00	(—)	—	312	27.9	1.00	(—)	—
Black	334	19.4	225	67.4	1.12	(0.86 – 1.45)	0.43	96	28.7	1.04	(0.79 – 1.37)	0.82
Indian	183	10.6	114	62.3	0.90	(0.65 – 1.24)	0.56	53	29.0	1.05	(0.75 – 1.49)	0.84
Other	87	5.1	58	66.7	1.08	(0.68 – 1.72)	0.82	23	26.4	0.93	(0.57 – 1.52)	0.86
Maternal age (categories) *N=1718*												
<20 years	157	9.1	98	62.4	1.00	(—)	—	50	31.8	1.00	(—)	—
20-24 years	344	20.0	214	62.2	0.99	(0.67 – 1.46)	1.00	96	27.9	0.83	(0.55 – 1.25)	0.43
25-29 years	467	27.1	305	65.3	1.13	(0.78 – 1.65)	0.58	129	27.6	0.82	(0.55 – 1.21)	0.36
30-34 years	388	22.5	253	65.2	1.13	(0.77 – 1.66)	0.61	116	29.9	0.91	(0.61 – 1.36)	0.73

Continued on next page...

Table S1: (Continued) Descriptive statistics of explanatory variables in relation to condition at birth or to delivery room death in the complete population of babies born at 22-26 completed weeks of gestation in England in 2006 who were known to be alive at admission to hospital and at the start of labour monitoring or decision to perform Caesarean section.

**Variable (Levels)**	**N**	**(%)**	**Born in good condition**	**(HR %)**	**OR**	**(95% CI)**	**P-value**	**Peri-natal death**	**(%)**	**OR**	**(95% CI)**	**P-value**
35-39 years	278	16.1	193	69.4	1.37	(0.91 – 2.06)	0.17	73	26.3	0.76	(0.50 – 1.17)	0.26
>40 years	84	4.9	58	69.0	1.34	(0.76 – 2.36)	0.38	19	22.6	0.63	(0.34 – 1.15)	0.17
Gestational age at booking *N=1551*												
<10 weeks	463	26.9	295	63.7	1.00	(—)	—	136	29.4	1.00	(—)	—
10-12 weeks	487	28.3	314	64.5	1.03	(0.79 – 1.35)	0.86	142	29.2	0.99	(0.75 – 1.31)	1.00
13-19 weeks	487	28.3	320	65.7	1.09	(0.84 – 1.42)	0.57	137	28.1	0.94	(0.71 – 1.25)	0.73
20+ weeks	114	6.6	80	70.2	1.34	(0.86 – 2.09)	0.24	26	22.8	0.71	(0.44 – 1.15)	0.20
Maternal smoking *N=1722*												
no	1303	75.7	851	65.3	1.00	(—)	—	365	28.0	1.00	(—)	—
yes	419	24.3	271	64.7	0.97	(0.77 – 1.22)	0.86	119	28.4	1.02	(0.80 – 1.30)	0.93
Maternal body mass index (BMI) *N=1481*												
<18.5 (underweight)	56	3.3	31	55.4	1.00	(—)	—	17	30.4	1.00	(—)	—
18.5-24.9 (appropriate)	658	38.2	421	64.0	1.43	(0.83 – 2.48)	0.25	189	28.7	0.92	(0.51 – 1.67)	0.92
>=25 (overweight)	767	44.5	497	64.8	1.48	(0.86 – 2.57)	0.2	227	29.6	0.96	(0.53 – 1.74)	1.00
Pre-pregnancy diabetes, type I or II *N=1709*												
no/miss	1690	98.1	1104	65.3	1.00	(—)	—	476	28.2	1.00	(—)	—
yes	19	1.1	14	73.7	1.49	(0.53 – 4.15)	0.60	3	15.8	0.48	(0.14 – 1.65)	0.35
Non-insulin dependent gestational diabetes *N=1709*												
no/miss	1702	98.8	1114	65.5	1.00	(—)	—	477	28.0	1.00	(—)	—
yes	7	0.4	4	57.1	0.70	(0.16 – 3.15)	0.95	2	28.6	1.03	(0.2 – 5.31)	1.00
Essential hypertension, on treatment at booking *N=1709*												
no/miss	1656	96.2	1080	65.2	1.00	(—)	—	467	28.2	1.00	(—)	—
yes	53	3.1	38	71.7	1.35	(0.74 – 2.48)	0.41	12	22.6	0.75	(0.39 – 1.43)	0.46
Epilepsy *N=1709*												
no/miss	1697	98.5	1110	65.4	1.00	(—)	—	476	28.0	1.00	(—)	—
yes	12	0.7	8	66.7	1.06	(0.32 – 3.53)	1.00	3	25.0	0.86	(0.23 – 3.17)	1.00
Other important medical history *N=1709*												
no/miss	1694	98.4	1107	65.3	1.00	(—)	—	477	28.2	1.00	(—)	—
yes	15	0.9	11	73.3	1.46	(0.46 – 4.60)	0.71	2	13.3	0.39	(0.09 – 1.75)	0.33
Any maternal medical problems *N=1711*												
no	1610	93.5	1046	65.0	1.00	(—)	—	458	28.4	1.00	(—)	—
yes	101	5.9	72	71.3	1.34	(0.86 – 2.08)	0.24	21	20.8	0.66	(0.40 – 1.08)	0.12
Parity (categorical) *N=774*												
Primip	365	21.2	244	66.8	1.00	(—)	—	98	26.8	1.00	(—)	—

Continued on next page...

Table S1: (Continued) Descriptive statistics of explanatory variables in relation to condition at birth or to delivery room death in the complete population of babies born at 22-26 completed weeks of gestation in England in 2006 who were known to be alive at admission to hospital and at the start of labour monitoring or decision to perform Caesarean section.

**Variable (Levels)**	**N**	**(%)**	**Born in good condition**	**(HR %)**	**OR**	**(95% CI)**	**P-value**	**Peri-natal death**	**(%)**	**OR**	**(95% CI)**	**P-value**
G1	344	20.0	215	62.5	0.83	(0.61 – 1.13)	0.26	90	26.2	0.97	(0.69 – 1.35)	0.9
2+ prev. babies	65	3.8	45	69.2	1.12	(0.63 – 1.97)	0.82	16	24.6	0.89	(0.48 – 1.64)	0.82
Primiparous (first registrable birth) *N=1701*												
no	858	49.8	560	65.3	1.00	(—)	—	228	26.6	1.00	(—)	—
yes	843	49.0	554	65.7	1.02	(0.84 – 1.25)	0.89	246	29.2	1.14	(0.92 – 1.41)	0.25
Primigravid (first pregnancy) *N=1712*												
No	1180	68.5	765	64.8	1.00	(—)	—	334	28.3	1.00	(—)	—
Yes	532	30.9	353	66.4	1.07	(0.86 – 1.33)	0.58	146	27.4	0.96	(0.76 – 1.2)	0.76
Previous Caesarean *N=1677*												
no	1445	83.9	951	65.8	1.00	(—)	—	402	27.8	1.00	(—)	—
yes	232	13.5	154	66.4	1.03	(0.76 – 1.38)	0.92	58	25.0	0.86	(0.63 – 1.19)	0.42
Previous premature birth *N=1687*												
no	1420	82.5	941	66.3	1.00	(—)	—	395	27.8	1.00	(—)	—
yes	267	15.5	168	62.9	0.86	(0.66 – 1.13)	0.32	71	26.6	0.94	(0.70 – 1.26)	0.74
IMD national quintiles *N=1700*												
Least	615	35.7	392	63.7	1.00	(—)	—	189	30.7	1.00	(—)	—
2	582	33.8	378	64.9	1.05	(0.83 – 1.34)	0.71	169	29.0	0.92	(0.72 – 1.18)	0.56
3	389	22.6	261	67.1	1.16	(0.89 – 1.52)	0.31	101	26.0	0.79	(0.59 – 1.05)	0.12
4	112	6.5	75	67.0	1.15	(0.75 – 1.77)	0.58	21	18.8	0.52	(0.31 – 0.86)	0.01
Most	2	0.1	2	100.0	∞	(0.00 – ∞)	0.74	0	0.0	0.00	(0.00 – ∞)	0.86
IMD study quintiles *N=1700*												
Least	532	30.9	336	63.2	1.00	(—)	—	163	30.6	1.00	(—)	—
2	506	29.4	326	64.4	1.06	(0.82 – 1.36)	0.72	151	29.8	0.96	(0.74 – 1.26)	0.83
3	412	23.9	278	67.5	1.21	(0.92 – 1.59)	0.19	111	26.9	0.83	(0.63 – 1.11)	0.24
4	214	12.4	146	68.2	1.25	(0.89 – 1.76)	0.22	49	22.9	0.67	(0.47 – 0.97)	0.04
Most	36	2.1	22	61.1	0.92	(0.46 – 1.83)	0.95	6	16.7	0.45	(0.18 – 1.11)	0.11
Any counselling *N=1722*												
No	227	13.2	132	58.1	1.00	(—)	—	67	29.5	1.00	(—)	—
Yes	1495	86.8	990	66.2	1.41	(1.06 – 1.88)	0.02	417	27.9	0.92	(0.68 – 1.26)	0.67
Were any options discussed *N=1722*												
No	773	44.9	558	72.2	1.00	(—)	—	149	19.3	1.00	(—)	—
Yes	949	55.1	564	59.4	0.56	(0.46 – 0.69)	< 0.001	335	35.3	2.28	(1.83 – 2.86)	< 0.001
Paediatric counselling *N=1633*												
no	387	22.5	216	55.8	1.00	(—)	—	136	35.1	1.00	(—)	—

Continued on next page...

Table S1: (Continued) Descriptive statistics of explanatory variables in relation to condition at birth or to delivery room death in the complete population of babies born at 22-26 completed weeks of gestation in England in 2006 who were known to be alive at admission to hospital and at the start of labour monitoring or decision to perform Caesarean section.

**Variable (Levels)**	**N**	**(%)**	**Born in good condition**	**(HR %)**	**OR**	**(95% CI)**	**P-value**	**Peri-natal death**	**(%)**	**OR**	**(95% CI)**	**P-value**
yes	1246	72.4	846	67.9	1.67	(1.33 – 2.11)	< 0.001	326	26.2	0.65	(0.51 – 0.83)	< 0.001
Choice made about newborn care (resus/IC) *N=1235*												
none expressed	508	29.5	336	66.1	1.00	(—)	—	131	25.8	1.00	(—)	—
full NIC	391	22.7	309	79.0	1.93	(1.42 – 2.62)	< 0.001	44	11.3	0.36	(0.25 – 0.53)	< 0.001
withhold IC	45	2.6	5	11.1	0.06	(0.02 – 0.17)	< 0.001	43	95.6	61.87	(14.78 – 258.98)	< 0.001
paed discretion	291	16.9	139	47.8	0.47	(0.35 – 0.63)	< 0.001	140	48.1	2.67	(1.97 – 3.62)	< 0.001
Decision not to perform C/S for fetal distress *N=1158*												
No	807	46.9	578	71.6	1.00	(—)	—	169	20.9	1.00	(—)	—
Yes	351	20.4	175	49.9	0.39	(0.30 – 0.51)	< 0.001	158	45.0	3.09	(2.36 – 4.05)	< 0.001
Withholding care discussed *N=1178*												
No	713	41.4	539	75.6	1.00	(—)	—	106	14.9	1.00	(—)	—
Yes	465	27.0	218	46.9	0.28	(0.22 – 0.37)	< 0.001	239	51.4	6.06	(4.60 – 7.97)	< 0.001
Senior obstetric counselling *N=1722*												
No	435	25.3	270	62.1	1.00	(—)	—	120	27.6	1.00	(—)	—
Yes	1287	74.7	852	66.2	1.2	(0.96 – 1.50)	0.13	364	28.3	1.04	(0.81 – 1.32)	0.83
PROM >24 hrs *N=1710*												
no	1211	70.3	793	65.5	1.00	(—)	—	344	28.4	1.00	(—)	—
yes	499	29.0	324	64.9	0.98	(0.78 – 1.21)	0.87	136	27.3	0.94	(0.75 – 1.19)	0.67
Abruption *N=1710*												
no	1577	91.6	1043	66.1	1.00	(—)	—	428	27.1	1.00	(—)	—
yes	133	7.7	74	55.6	0.64	(0.45 – 0.92)	0.02	52	39.1	1.72	(1.20 – 2.48)	< 0.001
APH >20 weeks *N=1710*												
no	1406	81.6	916	65.1	1.00	(—)	—	393	28	1.00	(—)	—
yes	304	17.7	201	66.1	1.04	(0.80 – 1.36)	0.80	87	28.6	1.03	(0.79 – 1.36)	0.87
Pre-eclampsia *N=1710*												
no	1586	92.1	1014	63.9	1.00	(—)	—	465	29.3	1.00	(—)	—
yes	124	7.2	103	83.1	2.77	(1.71 – 4.47)	< 0.001	15	12.1	0.33	(0.19 – 0.58)	< 0.001
Cervical suture *N=1710*												
no	1613	93.7	1049	65.0	1.00	(—)	—	460	28.5	1.00	(—)	—
yes	97	5.6	68	70.1	1.26	(0.81 – 1.97)	0.36	20	20.6	0.65	(0.39 – 1.08)	0.12
Obstetric complication in pregnancy *N=1712*												
no	723	42	454	62.8	1.00	(—)	—	219	30.3	1.00	(—)	—
yes	989	57.4	663	67.0	1.21	(0.99 – 1.47)	0.08	261	26.4	0.83	(0.67 – 1.02)	0.09

Continued on next page...

Table S1: (Continued) Descriptive statistics of explanatory variables in relation to condition at birth or to delivery room death in the complete population of babies born at 22-26 completed weeks of gestation in England in 2006 who were known to be alive at admission to hospital and at the start of labour monitoring or decision to perform Caesarean section.

**Variable (Levels)**	**N**	**(%)**	**Born in good condition**	**(HR %)**	**OR**	**(95% CI)**	**P-value**	**Peri-natal death**	**(%)**	**OR**	**(95% CI)**	**P-value**
Fetal complication *N=1695*												
none	1545	89.7	1005	65	1.00	(—)	—	435	28.2	1.00	(—)	—
iugr	91	5.3	68	74.7	1.59	(0.98 – 2.58)	0.08	19	20.9	0.67	(0.40 – 1.13)	0.17
oligo	38	2.2	22	57.9	0.74	(0.38 – 1.42)	0.46	13	34.2	1.33	(0.67 – 2.62)	0.52
both	21	1.2	15	71.4	1.34	(0.52 – 3.48)	0.70	6	28.6	1.02	(0.39 – 2.65)	1.00
Chorioamnionitis - definite or suspected *N=1683*												
no	1285	74.6	836	65.1	1.00	(—)	—	355	27.6	1.00	(—)	—
yes	398	23.1	262	65.8	1.03	(0.82 – 1.31)	0.82	119	29.9	1.12	(0.87 – 1.43)	0.41
Labour type (binary) *N=1712*												
none	292	17.0	233	79.8	1.00	(—)	—	48	16.4	1.00	(—)	—
spont	1420	82.5	884	62.3	0.42	(0.31 – 0.57)	< 0.001	434	30.6	2.24	(1.61 – 3.11)	< 0.001
Tocolysis (by type) *N=1708*												
None	1283	74.5	782	61.0	1.00	(—)	—	423	33.0	1.00	(—)	—
Atosiban	143	8.3	118	82.5	3.02	(1.94 – 4.72)	< 0.001	14	9.8	0.22	(0.13 – 0.39)	< 0.001
Ritodrine	7	0.4	6	85.7	3.84	(0.46 – 32.02)	0.34	1	14.3	0.34	(0.04 – 2.82)	0.52
Indometacin	27	1.6	24	88.9	5.13	(1.54 – 17.11)	0.01	2	7.4	0.16	(0.04 – 0.69)	0.01
Nifedipine	189	11	140	74.1	1.83	(1.30 – 2.58)	< 0.001	33	17.5	0.43	(0.29 – 0.64)	< 0.001
Other	19	1.1	16	84.2	3.42	(0.99 – 11.79)	0.07	1	5.3	0.11	(0.02 – 0.85)	0.02
More than one	40	2.3	31	77.5	2.21	(1.04 – 4.67)	0.05	6	15.0	0.36	(0.15 – 0.86)	0.03
Antenatal (in utero) transfer *N=1705*												
no	1370	79.6	844	61.6	1.00	(—)	—	434	31.7	1.00	(—)	—
yes	335	19.5	276	82.4	2.92	(2.16 – 3.94)	< 0.001	39	11.6	0.28	(0.20 – 0.40)	< 0.001
Maternal antibiotics in labour *N=1696*												
none	881	51.2	581	65.9	1.00	(—)	—	248	28.1	1.00	(—)	—
proph	468	27.2	309	66.0	1.00	(0.79 – 1.27)	1.00	122	26.1	0.90	(0.70 – 1.16)	0.45
treatment	347	20.2	217	62.5	0.86	(0.67 – 1.12)	0.29	108	31.1	1.15	(0.88 – 1.51)	0.33
Presentation *N=1643*												
Cephalic	915	53.1	657	71.8	1.00	(—)	—	199	21.7	1.00	(—)	—
Breech	653	37.9	379	58.0	0.54	(0.44 – 0.67)	< 0.001	234	35.8	2.01	(1.61 – 2.51)	< 0.001
Other	75	4.4	47	62.7	0.66	(0.40 – 1.08)	0.12	21	28	1.40	(0.83 – 2.37)	0.27
Unit NHS category *N=1693*												
1	147	8.5	70	47.6	0.35	(0.25 – 0.50)	< 0.001	69	46.9	3.31	(2.31 – 4.74)	< 0.001
2	583	33.9	351	60.2	0.58	(0.47 – 0.73)	< 0.001	201	34.5	1.97	(1.56 – 2.48)	< 0.001
3	963	55.9	695	72.2	1.00	(—)	—	203	21.1	1.00	(—)	—

[a]Chi-squared test for trend: < 0.001 ;
[b]Chi-squared test for trend: 0.049 ;
[c]Chi-squared test for trend: 0.528 ;
[d]Level 1: Special Care Baby Unit; Level 2: Local Neonatal Unit; Level 3: Network Intensive Care Unit.

Supplementary table 2 — Steroids sensitivity analysis results

Table S2: Odds ratios of the effect of partial or full course of steroids on the presence of a heart rate greater than 100 at 5 minutes of age or to delivery room death in babies at 22-26 completed weeks of gestation to the population of women who received counselling in England in 2006.

Model	N	Partial course OR	Partial course (95% CI)	Full course OR	Full course (95% CI)	P-value
**Condition at birth**						
Baseline	1484	5.43	(3.98 – 7.42)	8.32	(6.25 – 11.08)	< 0.001
Baseline + GA [a]	1484	2.05	(1.41 – 2.98)	2.65	(1.85 – 3.80)	< 0.001
Clinical [b]	1423	1.76	(1.18 – 2.62)	2.18	(1.47 – 3.23)	< 0.001
Complete [c]	1423	1.56	(1.02 – 2.37)	2.18	(1.43 – 3.33)	< 0.001
Models with effect modification by mode of delivery:						
Clinical (vaginal delivery) [b]	1423	2.11	(1.38 – 3.23)	2.23	(1.45 – 3.41)	0.023
Clinical (caesarean delivery) [b]		0.53	(0.17 – 1.65)	1.47	(0.48 – 4.46)	
Complete (vaginal delivery) [c]	1423	1.88	(1.20 – 2.94)	2.27	(1.44 – 3.57)	0.029
Complete (caesarean delivery) [c]		0.45	(0.14 – 1.46)	1.33	(0.42 – 4.20)	
**Delivery Room death**						
Baseline	1484	0.12	(0.08 – 0.16	0.07	(0.06 – 0.10)	< 0.001
Baseline + GA [a]	1484	0.38	(0.25 – 0.58)	0.32	(0.22 – 0.48)	< 0.001
Clinical [b]	1423	0.45	(0.29 – 0.69)	0.36	(0.23 – 0.56)	< 0.001
Complete [c]	1423	0.53	(0.33 – 0.86)	0.35	(0.22 – 0.58)	< 0.001
Models with effect modification by mode of delivery:						
Clinical (vaginal delivery) [b]	1423	0.38	(0.24 – 0.60)	0.33	(0.20 – 0.52)	0.056
Clinical (caesarean delivery) [b]		2.48	(0.47 – 12.97)	1.04	(0.21 – 5.16)	
Complete (vaginal delivery) [c]	1423	0.44	(0.26 – 0.73)	0.31	(0.18 – 0.52)	0.033
Complete (caesarean delivery) [c]		4.91	(0.63 – 38.35)	2.09	(0.28 – 15.47)	

[a] GA: gestational age
[b] The clinical models were additionally adjusted for: mode of delivery, *in utero* transfer, maternal pre-eclampsia and placental abruption.
[c] The complete models were adjusted for the same factors as the clinical models, plus: who the parents were counselled by, whether a decision was made not to perform caesarean section in cases of fetal distress, what choice was expressed for resuscitation, and whether withholding care was discussed.

Supplementary table 3 — Sensitivity analysis results for mode of delivery

Table S3: Odds ratios of the effect of delivery by caesaerean section on the presence of a heart rate greater than 100 at 5 minutes of age or to delivery room death in the population of babies born at 22-26 completed weeks of gestation to women who received a partial or full course of antenatal steroids in England in 2006.

Model	N	OR	(95% CI)	P-value
**Condition at birth**				
Baseline	1213	2.31	(1.61 – 3.31)	<0.001
Baseline + GA [a]	1213	1.46	(0.99 – 2.15)	<0.001
Models with effect modification by labour type:				
No labour [b]	1212	4.08	(1.96 – 8.50)	0.003
Spont labour [b]		0.86	(0.52 – 1.43)	
Clinical: no labour [c]	1172	8.67	(3.47 – 21.70)	0.0001
Clinical: spont labour [c]		1.22	(0.71 – 2.09)	
Complete: no labour [d]	1172	5.55	(2.13 – 14.47)	0.016
Complete: spont labour [d]		1.03	(0.60 – 1.79)	
**Delivery Room death**				
Baseline	1213	0.38	(0.24 – 0.61)	<0.001
Baseline + GA [a]	1213	0.84	(0.51 – 1.39)	<0.001
Models with effect modification by labour type:				
No labour [b]	1212	0.19	(0.08 – 0.46)	0.0002
Spont labour [b]		1.47	(0.76 – 2.82)	
Clinical: no labour [e]	1178	0.05	(0.02 – 0.17)	<0.001
Clinical: spont labour [e]		0.90	(0.45 – 1.81)	
Complete: no labour [f]	1210	0.10	(0.03 – 0.34)	0.033
Complete: spont labour [f]		1.18	(0.58 – 2.43)	

[a] GA: gestational age. [b] Adjusted for gestational age.
[c] Adjusted for: GA, labour type,*in utero* transfer, presentation at delivery, NICU level at birth hospital, maternal pre-eclampsia and placental abruption.
[d] Adjusted for the same factors as the clinical model ('c'), plus: who the parents were counselled by, whether a decision was made not to perform Caesarean section in cases of fetal distress, and what choice was expressed for resuscitation. [e] Adjusted for: GA, labour type, antenatal steroids, presentation at delivery, maternal pre-eclampsia and placental abruption. [f] Adjusted for the same factors as the clinical model ('e'), plus: who the parents were counselled by, whether a decision was made not to perform Caesarean section in cases of fetal distress, what choice was expressed for resuscitation, and whether withholding care was discussed.

Author details

[1]Institute for Womens' Health, UCL, 74 Huntley Street, WC1E 6AU, London, UK. [2] Homerton Hospital, London, UK. [3] University of Leicester, Leicester, UK. [4] University of Liverpool, Liverool, UK. [5] The Wolfson Institute, Queen Mary University of London, London, UK.

References
