# Supplementary material for: Impact of obstetric interventions on condition at birth in extremely preterm babies: evidence from a national cohort study
Source: BMC Pregnancy Childbirth. 2016 Dec 13;16:390. doi: 10.1186/s12884-016-1154-y (PMC5154160; doi:10.1186/s12884-016-1154-y)
Supplement: Additional file 3 — STROBE Statement checklist. (PDF 67 KB) [file 12884_2016_1154_MOESM3_ESM.pdf]

RESEARCH

Additional file 3: STROBE Checklist

Andrei S Morgan[1*], Neil Marlow[1], Elizabeth S Draper[3], Zarko Alfirević[4], Enid M Hennessy[5] and Kate Costeloe[2]

*Correspondence:
andrei.morgan@ucl.ac.uk
[1]Institute for Womens' Health, UCL, 74 Huntley Street, WC1E 6AU, London, UK
Full list of author information is available at the end of the article

1 STROBE Statement checklist

The following table contains the STROBE checklist of items to be included in reports of cohort studies alongside a reference to where in the article the information may be found.

STROBE checklist for "Impact of obstetric interventions on condition at birth in extremely preterm babies: evidence from a national cohort study"

	Item No	Recommendation	Page (notes)
**Title and abstract**	1	(a) Indicate the study's design with a commonly used term in the title or the abstract	Page 1 (Title)
		(b) Provide in the abstract an informative and balanced summary of what was done and what was found	Page 1
**Introduction**			
Background/rationale	2	Explain the scientific background and rationale for the investigation being reported	Page 2 (background section)
Objectives	3	State specific objectives, including any prespecified hypotheses	Page 2 (final paragraph of background)
**Methods**			
Study design	4	Present key elements of study design early in the paper	Page 2 (methods)
Setting	5	Describe the setting, locations, and relevant dates, including periods of recruitment, exposure, follow-up, and data collection	Page 2 (methods)
Participants	6	(a) Give the eligibility criteria, and the sources and methods of selection of participants. Describe methods of follow-up	Page 2 (methods)
		(b) For matched studies, give matching criteria and number of exposed and unexposed	N/A
Variables	7	Clearly define all outcomes, exposures, predictors, potential confounders, and effect modifiers. Give diagnostic criteria, if applicable	Page 3 (study variables)
Data sources/measurement	8[a]	For each variable of interest, give sources of data and details of methods of assessment (measurement). Describe comparability of assessment methods if there is more than one group.	Pages 2 (methods) and 3 (study variables)

Continued on next page...

Strobe checklist (continued)

	Item No	Recommendation	Page (notes)
Bias	9	Describe any efforts to address potential sources of bias	Page 4 (final paragraph of 'statistical methods')
Study size	10	Explain how the study size was arrived at	Pages 2 (methods) and Additional file 1: Figure 1. Additional information relating to this is provided in the other figures (figure 1, page 17, and Additional file 1: Figures 2-4)
Quantitative variables	11	Explain how quantitative variables were handled in the analyses. If applicable, describe which groupings were chosen and why	Page 3 (study variables)
Statistical methods	12	(a) Describe all statistical methods, including those used to control for confounding	Page 4 (statistical methods)
		(b) Describe any methods used to examine subgroups and interactions	Page 4 (statistical methods, second paragraph)
		(c) Explain how missing data were addressed	Page 5 (third paragraph)
		(d) If applicable, explain how loss to follow-up was addressed	N/A
		(e) Describe any sensitivity analyses	Page 4 (third paragraph)
**Results**			
Participants	13[a]	(a) Report numbers of individuals at each stage of study—eg numbers potentially eligible, examined for eligibility, confirmed eligible, included in the study, completing follow-up, and analysed	Page 4 (Results: "population" section) and Additional file 1: Figure 1. Additional information relating to this is provided in the other figures (figure 1, page 15, and Additional file 1: figures 2-4)
		(b) Give reasons for non-participation at each stage	Additional file 1: Figure 1
		(c) Consider use of a flow diagram	Figure 1 (pages 17) and Additional file 1: figures 1-4
Descriptive data	14[a]	(a) Give characteristics of study participants (eg demographic, clinical, social) and information on exposures and potential confounders	Pages 4 (Results: "population" section), 6 (table 1) and Additional file 2: Table 1
		(b) Indicate number of participants with missing data for each variable of interest	Pages 4 (Results: "population" section), 6 (table 1) and Additional file 2: Table 1
		(c) Summarise follow-up time (eg, average and total amount)	N/A
Outcome data	15[a]	Report numbers of outcome events or summary measures over time	Page 5 (Results: "population" section, third paragraph) and page 6 (table 1)
Main results	16	(a) Give unadjusted estimates and, if applicable, confounder-adjusted estimates and their precision (eg, 95% confidence interval). Make clear which confounders were adjusted for and why they were included	Pages 4-11 (Entire "results" section. Unadjusted results are presented first, with confounder adjusted estimates listed under each of the main exposures: antenatal steroids, tocolysis and mode of delivery)
		(b) Report category boundaries when continuous variables were categorized	Additional file 2: Table 1
		(c) If relevant, consider translating estimates of relative risk into absolute risk for a meaningful time period	N/A

Continued on next page...

Strobe checklist (continued)

	Item No	Recommendation	Page (notes)
Other analyses	17	Report other analyses done—eg analyses of subgroups and interactions, and sensitivity analyses	Pages 8-11 (sensitivity analyses, discussed under each of the respective exposures)
**Discussion**			
Key results	18	Summarise key results with reference to study objectives	Page 1 (principal findings)
Limitations	19	Discuss limitations of the study, taking into account sources of potential bias or imprecision. Discuss both direction and magnitude of any potential bias	Pages 11-13 (Strengths and limitations of this study)
Interpretation	20	Give a cautious overall interpretation of results considering objectives, limitations, multiplicity of analyses, results from similar studies, and other relevant evidence	Pages 13-14 (Study findings in context)
Generalisability	21	Discuss the generalisability (external validity) of the study results	Pages 13-14 (Study findings in context)
**Other information**			
Funding	22	Give the source of funding and the role of the funders for the present study and, if applicable, for the original study on which the present article is based	Page 14 (Funding)

a Give information separately for exposed and unexposed groups.

Note: An Explanation and Elaboration article discusses each checklist item and gives methodological background and published examples of transparent reporting. The STROBE checklist is best used in conjunction with this article (freely available on the Web sites of PLoS Medicine at http://www.plosmedicine.org/, Annals of Internal Medicine at http://www.annals.org/, and Epidemiology at http://www.epidem.com/). Information on the STROBE Initiative is available at http://www.strobe-statement.org.

Author details

[1]Institute for Womens' Health, UCL, 74 Huntley Street, WC1E 6AU, London, UK. [2] Homerton Hospital, London, UK. [3] University of Leicester, Leicester, UK. [4] University of Liverpool, Liverool, UK. [5] The Wolfson Institute, Queen Mary University of London, London, UK.

References
